# Regional Differences in Medicare Reimbursements and Gastroenterology Workforce Dynamics: Implications for Access to Care

**DOI:** 10.3390/healthcare14020267

**Published:** 2026-01-21

**Authors:** Jason N. Chen, Eric C. H. Leung, Jacob Evans, Cassidy Swain, Arham Siddiqui, Duke Appiah, Sameer Islam

**Affiliations:** 1 Department of Internal Medicine, The University of Texas Health Science Center, San Antonio, TX 78229, USA; leunge@uthscsa.edu (E.C.H.L.); cassidy.swn@gmail.com (C.S.); siddiquia1@uthscsa.edu (A.S.); 2 School of Medicine, Texas Tech University Health Sciences Center, Lubbock, TX 79430, USA; jacob.t.evans@ttuhsc.edu (J.E.); duke.appiah@ttuhsc.edu (D.A.); sislam@lubbockgastro.com (S.I.)

**Keywords:** Medicare reimbursement, aging population, GI workforce, health equity, health economics

## Abstract

**Background:** As the U.S. population ages, the need for gastrointestinal (GI) care and procedures grows. Medicare is a significant payer for these procedures, but declining reimbursements raise concerns about the availability of GIs and thus equitable access to care. This study examines the relationship between Medicare reimbursements for GI procedures and the regional supply and demand of GI physicians. **Methods:** This study analyzed the Medicare facility and non-facility setting physician reimbursements for the top 10 GI procedures for 2003, 2013, and 2023. Facility reimbursements were compared across four regions (Northeast, Midwest, South, and West) and compared to regional GI physician supply and demand data for 2013 and 2025 projections. Linear regression and mixed-effects models were used to evaluate relationships between reimbursements, physician supply, and demand. **Results:** The national average adjusted facility setting physician reimbursements for the top 10 GI procedures declined by 45.6% from 2003 to 2023. In 2013 and projected for 2025, the South had the highest GI physician supply and demand, but consistently lower facility setting physician reimbursements compared to the Northeast and West. Associations between supply, demand, and reimbursements were observed, though regional patterns showed paradoxical trends, such as similar low reimbursements in the South and Midwest despite differing supply levels. **Conclusions:** Regional inconsistencies between physician supply and reimbursements highlight the complexity of economic and healthcare dynamics. Declining Medicare reimbursements for GI procedures are multifactorial and, as the aging population grows, these reductions may widen disparities. Further investigation is needed to address barriers and ensure equitable access to GI care.

## 1. Introduction

Gastroenterologists (GIs) play an important role in providing screening, diagnostic, and therapeutic procedures. As the United States population ages, there is an increased need for these procedures to detect and treat gastrointestinal diseases in a demographic with increased incidence of patients with GI disorders who already face challenges in receiving equitable access to care [1,2].

With population aging, the number of Medicare beneficiaries also has increased, reaching a total enrollment of 67.3 million people [3], encompassing a significant proportion of GI physician reimbursements. Medicare reimburses physicians via Current Procedural Terminology (CPT) codes consisting of physician work, practice expense, and liability relative value units (RVUs) adjusted for geographic location and multiplied by a conversion rate. Many factors influence the reimbursement rate, leading to constant change and variation in reimbursements by geographic region. It is unclear how these changes affect availability of GI physicians and therefore patient access to GI care. Recent data further underscore these concerns, demonstrating that declining procedural reimbursements are associated with reductions in procedural volumes and fewer gastroenterologists performing endoscopic procedures [4].

Nationally, there has been a steady decrease in adjusted Medicare reimbursement rates for GI procedures [5]. While the effects of these reimbursements on GI physician supply and demand are unclear, they can have multiple downstream implications for healthcare access in regions with significant GI shortages. A 2025 analysis found that more than two-thirds of the United States counties lack a practicing gastroenterologist, and over 45 million Americans live more than 25 miles from the nearest gastroenterologist [6]. Counties that did not have gastroenterologists were more likely to be rural, lower-income, and have an older population, highlighting the substantial geographic disparities in access to gastroenterologists [6].

Structural and socioeconomic factors, including regional income levels, rurality, healthcare infrastructure, insurance composition, and population age distribution, play a substantial role in shaping both the gastroenterology workforce and reimbursement patterns. Regions with lower practice expense indices, higher poverty levels, and limited specialty infrastructure often receive systematically lower Medicare payments despite facing higher disease burden or fewer available clinicians, which is a pattern driven in part by geographic adjustments embedded within Medicare’s payment formula and the uneven distribution of social and economic resources across U.S. regions [7,8]. Additionally, county-level analyses show that social determinants of health, such as income and unemployment, explain substantial variation in Medicare spending, underscoring the structural inequities that influence healthcare financing and resource allocation [9]. These forces also shape physician distribution, with rural and socioeconomically disadvantaged areas more likely to experience GI shortages and reduced specialty access [4,6].

Previous studies have shown an inverse relationship between Medicare reimbursements and the number of primary care physicians in an area [10,11]. With the demand for GI physicians expected to outpace supply [12], the combination of this growing shortage and an increase in GI conditions among an aging population is concerning. Therefore, more detailed analysis on how Medicare reimbursements affect the supply and demand for GI physicians is essential to identifying deficiencies in providing equitable care. The aim of this investigation is to explore the relationship between Medicare reimbursements for the most common GI procedures and the regional supply and demand of GI physicians.

## 2. Methods

This cross-sectional study utilized the top 10 most frequently performed GI procedures using data from the American Society for Gastrointestinal Endoscopy and the American Gastroenterological Association [13]. The decision to focus on the top 10 GI procedures was based on their consistently high national utilization, frequent reporting in professional society analyses, and prior use as benchmark indicators in reimbursement evaluations [5]. Although gastroenterology is procedurally heterogeneous, these procedures represent a substantial proportion of real-world diagnostic and therapeutic volume and thus serve as a practical and representative proxy for evaluating reimbursement trends and workforce dynamics. Each procedure was matched with its corresponding Current Procedural Terminology (CPT) code, a universal standard for medical billing and documentation. The study was conducted with public data published by the Centers for Medicare and Medicaid Services with no patient identifiers; therefore, approval from the Institutional Review Board was unnecessary.

Using the Centers for Medicare and Medicaid Services (CMS) Physician Fee Schedule Look-Up Tool, we collected facility and non-facility setting pay rates for each procedure across all U.S. states for the years 2003, 2013, and 2023. The years 2003, 2013, and 2023 were selected to provide evenly spaced intervals across two decades while also utilizing the most recent year of complete reimbursement data available from CMS at the time of analysis. Using these time points allowed the evaluation of patterns over time and building upon prior work that examined reimbursement trends over similar periods [5]. Facility setting pay is the amount paid to physicians only for the professional component excluding the facility fees in a facility setting, such as hospitals and ambulatory surgery centers. Non-facility setting pay refers to physician reimbursements for both the professional service and the practice expenses in physician offices or other non-institutional settings. These payment amounts were determined using RVUs, geographic practice cost index, and a conversion factor. All payment amounts were adjusted for inflation and converted to January 2023 U.S. dollars using the U.S. Bureau of Labor Statistics’ Consumer Price Index (CPI), ensuring that trends reflect real purchasing power over time. The national adjusted facility and non-facility setting payments were compared between 2003, 2013, and 2023.

Additionally, the state facility setting pay were grouped into four geographic regions—Northeast, Midwest, West, and South—as defined by the Bureau of Health Workforce [12]. The physician reimbursement rates in facility settings for these regions were compared to regional gastroenterologist supply and demand figures for 2013 and for 2025 projections as reported by the Bureau of Health Workforce. Non-facility setting payments were not used for regional supply and demand analysis due to missing data from CMS.

### Statistical Analysis

General linear regression models were used to evaluate regional differences in the relation of the supply of gastroenterologists with reimbursements as well as the relation of reimbursement with the demand for gastroenterologists in cross-sectional analysis, with the exception of the projected data. In these separate regression models, reimbursement was the dependent variable while regional supply and demand were the independent variables. For analyses evaluating change in payment amounts from 2003 to 2023, and comparing regional differences in this change, linear mixed-effects models were used, with an unstructured covariance matrix selected by means of Akaike and Bayesian Information Criterion. For these models, change in reimbursement based on repeated measurements was the dependent variable while region and time were the independent variables. No adjustment for potential confounders was made as we used aggregate-level data. All analyses were performed using SAS software version 9.4 (SAS Institute), and *p* values less than 0.05 were used to determine statistical significance. Facility setting reimbursement data were complete. Non-facility setting reimbursement data had missing values across states, so these were not included in the supply and demand statistical analyses.

## 3. Results

**Figure 1:**
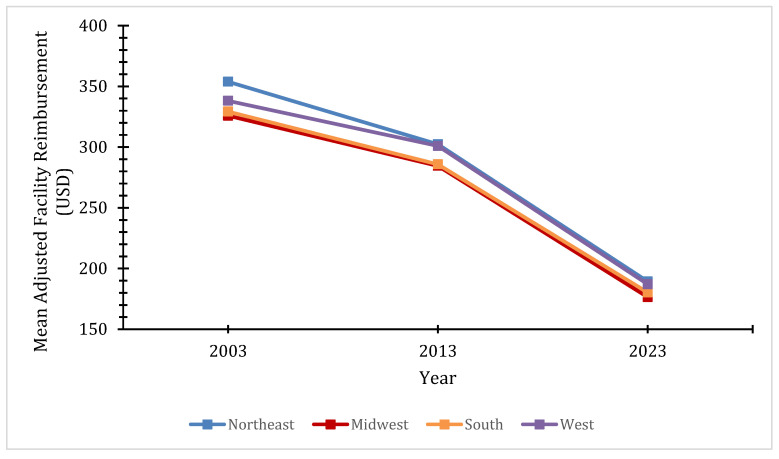
Regional mean adjusted facility setting physician reimbursements by year.

**Figure 2:**
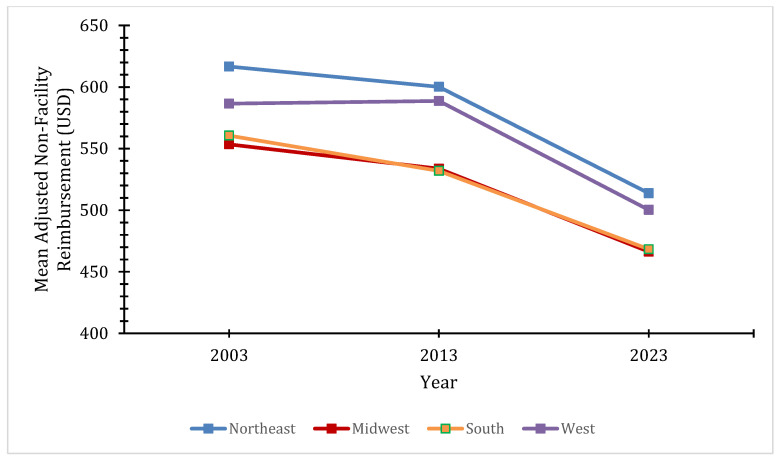
Regional mean adjusted non-facility setting physician reimbursements by year.

National average adjusted physician reimbursements in facility settings decreased 45.6% from USD 353.87 in 2003 to USD 189.28 in 2023. There was also an overall decrease in these reimbursements for every region, with significant inter-region variability (*p* = 0.003) (Figure 1). From 2003 to 2023, nationally, physician reimbursements in non-facility settings decreased by 15.9% (Figure 2). While varying decreases in these reimbursements within regions were observed, they were not statistically significant. Consistently, the Northeast had the highest reimbursements for both reimbursement types, followed closely by the West, while the South and Midwest consistently had the lowest.

The supply of GI physicians in 2013 and projected supply in 2025 are seen in Table 1. The southern region had the highest supply for GIs in 2013 and projected in 2025. Overall, there was an association between the supply of GI physicians and physician reimbursements in facility settings in 2013 (*p* = 0.03) (Table 1). The West with 2970 GIs and the Northeast with 3760 GIs had USD 15.20 (β = 15.2 (95% confidence interval (CI): 1.5 to 28.9)) and USD 16.61 (β = 16.6, 95% CI: 1.2 to 32.0) higher facility setting reimbursements, respectively, compared to the South, which demonstrated lower physician reimbursements but higher supply. The Midwest with the lowest supply of GIs did not have significantly different physician reimbursements than the South which had the greatest supply of GIs (β = −1.3, 95% CI: −15.3 to 12.8, *p* = 0.86). However, these two regions had the lowest average reimbursements of USD 284.51 and USD 285.78, respectively. Comparisons between 2023 reimbursements and 2025 physician supply are exploratory, as 2025 supply and demand data are projected estimates. In light of this, the Midwest (lowest projected supply) and the South (highest projected supply) had lower 2023 reimbursement levels than the Northeast and West (Table 1).

The demand for GI physicians in 2013 and the projected demand in 2025 are seen in Table 1. Overall, there was an association between the demand of GI physicians and physician reimbursements in facility settings in 2013 (*p* = 0.03). The southern region had the highest demand for GIs in 2013 and projected in 2025. Compared to the South that had the highest demand for GIs, the Northeast and West had significantly higher facility setting reimbursements (*p* = 0.03). Descriptively, facility setting reimbursements in 2023 were broadly similar across regions when viewed in the context of projected 2025 demand (Table 1).

## 4. Discussion

Over the past twenty years, adjusted Medicare reimbursements to physicians in both facility and non-facility settings for GI procedures have steadily declined across the nation and its regions. Nationally, changes in Medicare policies, such as adjustments to service volume, relative value units (RVUs), and the conversion factor, all driven by budget neutrality, have significantly contributed to the reduction in reimbursements [14]. Regionally, multiple factors have affected reimbursements, including the geographic index component of Medicare reimbursement calculations, demographic variations, differences in average allowed charges, and the proportion of beneficiaries meeting their deductibles [7]. Medicare adjusts for geographical variations through systems like the Hospital Wage Index (HWI) and Geographic Practice Cost Indexes (GPCI), which consider factors such as physician practice expenses, hospital labor cost, and professional liability insurance. However, these indices have been criticized for failing to accurately capture market value differences across regions [15]. Additionally, the zero-sum nature of Medicare’s budget neutrality policy means that any increase in reimbursement for one region necessitates cuts elsewhere, disproportionately affecting states with lower GPCI, particularly in the South [16]. Previous studies suggest that the clinical significance of diminishing reimbursements demonstrates inverse relationships between Medicare reimbursements and the number of primary care physicians in a region [10]. More recently, Busam and Shah found that declining procedural reimbursements were associated with reduced procedural volumes and fewer gastroenterologists continuing to perform endoscopic services, highlighting how reimbursement pressures may alter GI practice patterns [4].

Building off previous insights, our study further explores the nuanced relationship between the multifactorial intersection of Medicare facility reimbursements and GI physician supply and demand. First, regarding supply dynamics, our data presents an overall association between GI supply and 2013 facility setting reimbursement. Regarding demand, there is also an overall association between GI demand and 2013 facility setting reimbursement, which demonstrates more linear consistency and conventionally expected correlation between higher physician reimbursement and higher demand, specifically in northeast and west regions. Notably, when examining these same dynamics for projected 2023 data, these associations attenuate, suggesting an evolving and complex interaction between physician supply, demand, and reimbursement.

These results of overall associations between reimbursement and GI physician supply and demand that are influenced by regional analysis allude to nationally standardized physician fee schedules as the overall driver of GI physician supply and demand with region-specific factors attenuating the difference. Specifically, supply may holistically be reflective of national rather than regional patterns with regional variation more affected by non-financial factors such as training distribution, practice setting, healthcare density, lifestyle considerations, and regulation. Demand differences may be better explained by regional variation in clinical populations or resource-driven factors such as utilization, access, and capacity metrics suggesting price-inelasticity. This finding highlights the influence of region-specific variables and gaps in the complex interplay of economic and social factors in healthcare.

From a financial perspective, our data demonstrated an average variance of USD 15.37 in reimbursement. Although a precise estimate of gastroenterologist procedure volume is difficult to assess, best available evidence suggests three to thirteen procedures per day [17,18], with national studies estimating an average of 49 weeks worked per year [19]. Utilizing available estimates with our data, regional reimbursement differences account for a variation of USD 11,296 to USD 48,953 annually. However, this estimation provides limited insight into variation in annual reimbursement with unclear impacts on physician compensation.

Beyond economic metrics, patient outcomes are also shaped by harder-to-quantify factors, such as improvements in health and quality of life through early detection and intervention, psychological well-being from better access to care, and the avoidance of high-cost treatments due to early preventative screenings [20]. Supply and demand of GI physicians do not solely determine healthcare access, as factors like socioeconomic disadvantage through transportation challenges or food insecurity, lack of social support, higher rates of mental health disabilities, and low health literacy often play major roles in healthcare access and outcomes, particularly in the elderly, minority, and underserved communities [6,21]. Decreased payment rates may be associated with a higher risk of rural and underserved regions losing procedural clinics that depend on reimbursements [4]. Despite a current higher supply of GI physicians in the South, this region continues to experience lower Medicare reimbursements with growing demand, a combination that may contribute to widening disparities and existing geographic maldistribution, further limiting access to GI care in underserved communities.

Examining how reimbursement policies intersect with physician supply and demand provides an opportunity to advocate for more equitable healthcare reimbursement structures. This can be particularly beneficial for vulnerable populations, ensuring more balanced access to GI procedures and preventive care. Similar analysis in other subspecialty services has demonstrated that reduction in Medicare reimbursement disproportionately affects access to vascular and cardiac care in minority populations [22], raising concerns for extension into GI procedures. Decreased GI procedure access may disproportionately affect minority and economically disadvantaged populations who already experience higher morbidity and mortality of GI cancers and rely more on Medicare-based funding [23].

Ultimately, our findings underscore the complex and incongruent relationship between Medicare reimbursement, physician workforce distribution, and regional demand for GI services. While we identified overall associations between physician supply and demand with facility-based reimbursements, these relationships were inconsistent across regions and attenuated in projected data—suggesting that existing reimbursement structures inadequately reflect the realities of local healthcare delivery.

## 5. Limitations

This study has several limitations. Since the analysis is based on serial cross-sectional data, the findings reflect associations and cannot establish true temporal trajectories or causal relationships between reimbursement levels, physician supply, or clinical demand as aggregated data were used. Without access to granular data on physician billing practices and compensation models, comprehensive regional data on healthcare infrastructure, quality metrics, socioeconomic determinants, or actual clinical outcomes, our findings cannot establish how reimbursement variation impacts practice decisions, staffing, or patient care. Non-facility reimbursement data were excluded from regional analyses because CMS reporting was incomplete and inconsistent across states and years, which may underrepresent reimbursement patterns in office-based settings where non-facility billing is more common. An additional consideration is the focus on the top 10 most commonly performed GI procedures. Although this approach captures the majority of routine diagnostic and therapeutic volume and therefore offers good generalizability to common GI practice, it does not encompass less frequently performed or advanced procedures, which may limit applicability to certain tertiary or subspecialty settings.

## 6. Conclusions

The decline in Medicare reimbursements for GI procedures, together with regional differences in physician supply and demand, highlights the complex and uneven landscape in which GI services are delivered. Although associations were observed between reimbursement levels and workforce metrics, these patterns varied by region and were not consistently aligned, suggesting that reimbursement alone does not fully explain geographic disparities in GI access. As demand for GI care continues to grow, particularly in regions with longstanding shortages, further work is needed to understand how reimbursement structures interact with local workforce dynamics. While our study cannot determine causality, understanding how reimbursement patterns relate to regional supply and demand dynamics offers insight into potential contributors to geographic variation in access to care. Continued investigation is needed to clarify these relationships and to inform approaches that support more equitable availability of GI services across regions.

## Figures and Tables

**Table 1:** Comparison between 2013 regional supply and demand of gastroenterologists (GIs) and 2013 mean facility setting physician reimbursements as well as between projected 2025 regional supply and demand of GIs and 2023 mean facility setting physician reimbursements for the top 10 gastroenterology procedures.

Region	2013 Supply (n)	2013 Demand (n)	2013 Mean (95% CI) Facility Setting Physician Reimbursements (USD)	*p* Value	2025 Projected Supply (n)	2025 Projected Demand (n)	2023 Mean (95% CI) Facility Setting Physician Reimbursements (USD)
Northeast	3760	2840	302.4 (290.0–314.8)	0.03 *	3600	3060	189.3 (181.6–197.1)
Midwest	2780	3230	284.5 (273.8–295.3)	0.86	2690	3500	176.5 (169.8–183.2)
West	2970	3220	301.0 (290.6–311.3)	0.03 *	3560	4240	187.1 (180.6–193.5)
South	5090	5310	285.8 (276.7–294.8)	-	5690	6380	180.3 (174.7–185.9)

* *p* value testing regional differences is statistically significant from the South (*p* < 0.05). CI: confidence interval.

## Data Availability

Data was collected from the Centers for Medicare and Medicaid Services (CMS) Physician Fee Schedule Look-Up Tool found at https://www.cms.gov/medicare/physician-fee-schedule/search, accessed on 1 June 2024.
